# Supplementary material for: The interactome of intact mitochondria by cross-linking mass spectrometry provides evidence for coexisting respiratory supercomplexes
Source: Mol Cell Proteomics. 2017 Dec 8;17(2):216–32. doi: 10.1074/mcp.RA117.000470 (PMC5795388; doi:10.1074/mcp.RA117.000470)
Supplement: Supplemental Data [file supp_RA117.000470_133922_0_supp_23978_fzffwf.zip › spectra_annotation/mito_DR_spectra_annotation/203-2-4-1-6-1.pdf]

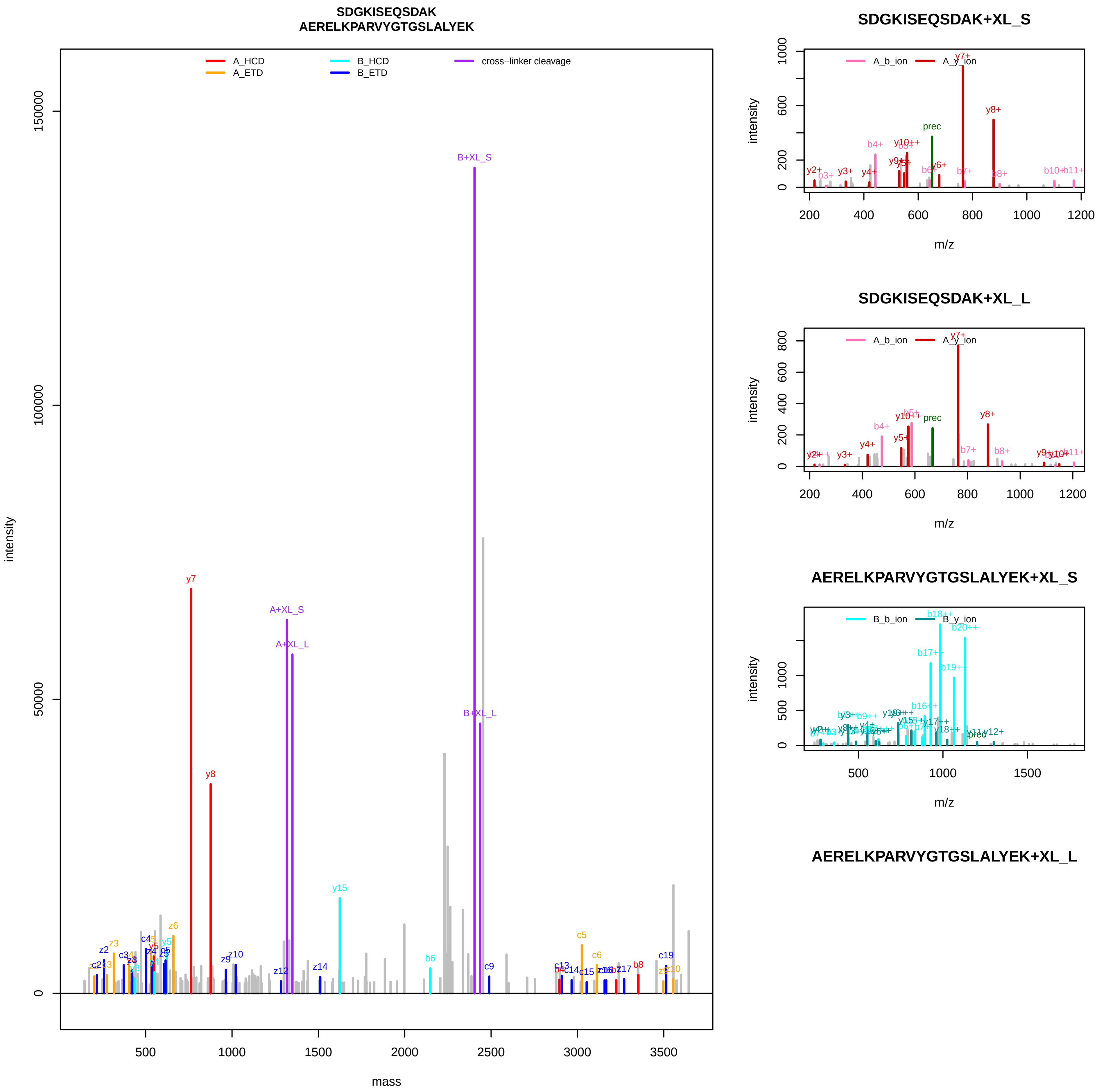

SDGKISEQSDAK
AERELKPARVYGTGSLALYEK
A_HCD
A_ETD
B_HCD
B_ETD
cross−linker cleavage
B+XL_S
A+XL_S
A+XL_L
B+XL_L
y7
y8
y15
b6
c9
c5
c6
b8
c19
z6
z10
z12
z14
intensity
mass
0
50000
100000
150000
500
1000
1500
2000
2500
3000
3500
SDGKISEQSDAK+XL_S
A_b_ion
A_y_ion
prec
y7+
y8+
b4+
y10++
m/z
intensity
SDGKISEQSDAK+XL_L
AERELKPARVYGTGSLALYEK+XL_S
B_b_ion
B_y_ion
b18++
b20++
b17++
b19++
b16++
AERELKPARVYGTGSLALYEK+XL_L
